# Complete genetic characterization of carbapenem-resistant *Acinetobacter johnsonii*, co-producing NDM-1, OXA-58, and PER-1 in a patient source

**DOI:** 10.3389/fcimb.2023.1227063

**Published:** 2023-08-25

**Authors:** Chongmei Tian, Jianqin Song, Lingzhi Ren, Delian Huang, Siwei Wang, Liping Fu, Yaping Zhao, Yongfeng Bai, Xueyu Fan, Tianhong Ma, Junjie Ying

**Affiliations:** ^1^ Department of Pharmacy, Shaoxing Hospital of Traditional Chinese Medicine Affiliated to Zhejiang Chinese Medical University, Shaoxing, Zhejiang, China; ^2^ Department of Traditional Chinese Medicine, Hangzhou Linping District Hospital of Integrated Chinese and Western Medicine, Hangzhou, China; ^3^ Department of Clinical Laboratory, The People’s Hospital of Zhangqiu Area, Jinan, China; ^4^ School of Medical Technology and Information Engineering, Zhejiang Chinese Medical University, Hangzhou, China; ^5^ Core Facility, The Quzhou Affiliated Hospital of Wenzhou Medical University, Quzhou People’s Hospital, Quzhou, China; ^6^ Department of Clinical Laboratory, The Quzhou Affiliated Hospital of Wenzhou Medical University, Quzhou People’s Hospital, Quzhou, China; ^7^ Department of Pharmacy, Jiaxing Hospital of Traditional Chinese Medicine, Jiaxing, China; ^8^ Department of Urology, The Quzhou Affiliated Hospital of Wenzhou Medical University, Quzhou People’s Hospital, Quzhou, China

**Keywords:** *Acinetobacter johnsonii*, carbapenem resistance, NDM-1, OXA-58, PER-1, integron

## Abstract

The emergence of carbapenemase-producing *Acinetobacter* spp. has been widely reported and become a global threat. However, carbapenem-resistant *A. johnsonii* strains are relatively rare and without comprehensive genetic structure analysis, especially for isolates collected from human specimen. Here, one *A. johnsonii* AYTCM strain, co-producing NDM-1, OXA-58, and PER-1 enzymes, was isolated from sputum in China in 2018. Antimicrobial susceptibility testing showed that it was resistant to meropenem, imipenem, ceftazidime, ciprofloxacin, and cefoperazone/sulbactam. Whole-genome sequencing and bioinformatic analysis revealed that it possessed 11 plasmids. *bla*_OXA-58_ and *bla*_PER-1_ genes were located in the pAYTCM-1 plasmid. Especially, a complex class 1 integron consisted of a 5′ conserved segment (5′ CS) and 3′ CS, which was found to carry sul1, arr-3, qnrVC6*, and bla*_PER-1_ cassettes. Moreover, the *bla*_NDM-1_ gene was located in 41,087 conjugative plasmids and was quite stable even after 70 passages under antibiotics-free conditions. In addition, six prophage regions were identified. Tracking of closely related plasmids in the public database showed that pAYTCM-1 was similar to pXBB1-9, pOXA23_010062, pOXA58_010030, and pAcsw19-2 plasmids, which were collected from the strains of sewage in China. Concerning the pAYTCM-3 plasmids, results showed that strains were collected from different sources and their hosts were isolated from various countries, such as China, USA, Japan, Brazil, and Mexico, suggesting that a wide spread occurred all over the world. In conclusion, early surveillance is warranted to avoid the extensive spread of this high-risk clone in the healthcare setting.

## Introduction

*Acinetobacter* spp. are ubiquitous in nature and are usually identified in the hospital environment, and some of these species have been reported in a variety of nosocomial infections (Wong et al., 2017). The most common species to cause infections is *A. baumannii*, followed by *A. calcoaceticus* and *A. lwoffii*. However, *A. johnsonii*, a kind of potentially opportunistic pathogen in *Acinetobacter* spp., generally distributed in natural or nosocomial environments, such as agricultural soil (Wang et al., 2019; Jia et al., 2021).

Carbapenems are the main antimicrobial agents for the treatment of infections with multidrug-resistant *Acinetobacter* spp., including *A. johnsonii* (Tang et al., 2020). However, the problem of carbapenem resistance is being increasingly reported, which has contributed to a huge challenge for clinicians (Bonnin et al., 2014). The carbapenem resistance mechanism was usually mediated via enzymatic inactivation (such as carbapenemases), efflux pump overexpression, and target site modification (i.e., altered penicillin-binding proteins) (Mohd Rani et al., 2017; Castanheira et al., 2023). Upon previous studies, more than 210 β-lactamases have been identified in *Acinetobacter* spp. with class D β-lactamases being the most widespread carbapenemase (Mohd Rani et al., 2017), including OXA-23, OXA-24, and OXA-58 (Liu et al., 2021). Moreover, several insertion sequence (IS) elements such as IS*Aba1* and IS*Aba3* could increase the expression of class D β-lactamase genes (including *bla*_OXA-58_-like and *bla*_OXA-23_-like genes) when they were found upstream of these IS elements (Mohd Rani et al., 2017).

Considering the increasing resistance to carbapenems and almost all other antimicrobial agents, *Acinetobacter* spp. are important resistant microorganisms with a global public health threat, which are associated with severe nosocomial infections including pneumonia, urinary tract, bloodstream, and wound infections (Gonzalez-Villoria and Valverde-Garduno, 2016). However, limited knowledge concerning the carbapenem resistance was known in *A. johnsonii* strains. Until now, researchers only reported some genome sequences and described the features of *A. johnsonii* strains which are isolated from the environment, especially in hospital sewage (Feng et al., 2016; Zong et al., 2020). However, little is known about this species which was collected from a patient source in the hospital. Here, we investigated the genetic characteristics of one carbapenem-resistant *A. johnsonii*, co-producing NDM-1, OXA-58, and PER-1 in a patient’s sputum in 2018 in China. To the best of our knowledge, this is the first comprehensive description of one carbapenem-resistant *A. johnsonii* from a patient source.

## Materials and methods

### Bacterial isolation and identification of the *A. johnsonii* AYTCM strain

A flowchart is shown in **Figure S1** (Behzadi and Gajdacs, 2021). *A. johnsonii* AYTCM strain was isolated from sputum in China in 2018. Isolate identification was conducted using matrix-assisted laser desorption ionization-time of flight mass spectrometry (MALDI-TOF MS, Bruker Daltonik GmbH, Bremen, Germany) and further confirmed by PCR and 16S rRNA (GenBank ID: NR_164627.1) gene-based sequencing with specific primers 27F (5′-agagtttgatcctggctcag-3′) and 1492R (5′-ggttaccttgttacgactt-3′) (Zong et al., 2020).

### Minimum inhibitory concentration measurement

Antimicrobial susceptibility testing (AST) was performed by the broth microdilution method and interpreted based on the recommendations of Clinical and Laboratory Standards Institute (CLSI) 2021 guidelines and European Committee on Antimicrobial Susceptibility Testing (EUCAST) 2021 breakpoint tables for tigecycline. The antimicrobial agents used in this study were shown as follows: ceftazidime (CAZ), cefoperazone/sulbactam (CFS), imipenem (IPM), meropenem (MEM), ciprofloxacin (CIP), amikacin (AMI), colistin (COL), tigecycline (TGC), and cefiderocol (CFDC). *Escherichia coli* ATCC 25922 served as the quality control strain.

### Mating experiments

To determine whether the plasmids carrying *bla*_NDM-1_, *bla*_OXA-58_, and *bla*_PER-1_ were transferable, conjugation experiments using *E. coli* J53 (sodium azide resistant) as the recipient strain were carried out using the filter mating method (Yang et al., 2021). Transconjugants were screened on Mueller–Hinton (MH) agar plates containing sodium azide (100 mg/L) and meropenem (2 mg/L). The identity of putative transconjugants was confirmed via PCR and MALDI-TOF MS.

### Stability experiments of plasmids carrying *bla*_NDM-1_, *bla*_OXA-58_, or *bla*_PER-1_ genes

*A. johnsonii* AYTCM strain was grown overnight at 37°C in 2 mL of Luria broth (LB) without antibiotics, followed by serial passage of 2-µL overnight culture into the 2-mL LB (1:1,000) each day, with a yield 10 generations, lasting for 7 days (Tian et al., 2022). On the last day, samples were collected and streaked onto antibiotic-free MHA plates. Colonies were selected randomly, and the presence of *bla*_NDM-1_, *bla*_OXA-58_, or *bla*_PER-1_ genes was confirmed by PCR with specific primers.

### Whole-genome sequencing, assembly, quality control, and annotation

Genomic DNA was extracted from *A. johnsonii* AYTCM strain using Qiagen Mini Kit (Qiagen, Germany) and Gentra^®^ Puregene^®^ Yeast/Bact. Kit (Qiagen, Germany) for Illumina and Nanopore sequencing, respectively. For trimming, quality control, and quality assessment of raw reads, fastp v 0.20.1 was used (Chen et al., 2018). *De novo* assembly of the reads of Illumina and MinION was constructed using Unicycler v0.4.8 (Wick et al., 2017). The assembly sequence was assessed via QUAST v 5.0.2 (Gurevich et al., 2013). Genome sequence annotation was conducted using the National Center for Biotechnology Information (NCBI) Prokaryotic Genome Annotation Pipeline (PGAP) (http://www.ncbi.nlm.nih.gov/genome/annotation_prok/) and the Rapid Annotation of microbial genomes using Subsystems Technology (RAST) server (Overbeek et al., 2014; Tatusova et al., 2016). Annotation function was further compared with *A. johnsonii* C6 (accession no. FUUY00000000) and MB44 (accession no. LBMO00000000) strains (Tian et al., 2016; Kaas et al., 2017).

### Bioinformatics analysis

Antimicrobial resistance genes were identified using the ABRicate program (https://github.com/tseemann/abricate) based on the ResFinder database (http://genomicepidemiology.org/) (Zankari et al., 2012). Bacterial virulence factors were identified using the virulence factor database (VFDB, http://www.mgc.ac.cn/VFs/) (Liu et al., 2022a). Average nucleotide identity (ANI) analysis with *A. johnsonii* C6 (accession no. FUUY00000000) and MB44 (accession no. LBMO00000000) strains was conducted using an ANI calculator (http://enve-omics.ce.gatech.edu/ani/index) (Luis and Konstantinos, 2016), and genome-based phylogenetic reconstruction with *A. johnsonii*, *A. baumannii*, *A. pittii*, and *A. seifertii* strains was further performed using the BacWGSTdb server (Marquez-Ortiz et al., 2017; Feng et al., 2021). Insertion sequences (ISs) were identified with ISfinder (Siguier et al., 2006). Conjugation transfer elements, including the origin site of DNA transfer (*oriT*), type IV secretion system (T4SS), type IV coupling protein (T4CP), and relaxase-related encoding genes, were predicted using *oriT*finder with default parameter settings (Li et al., 2018). PHAge Search Tool (PHAST) was utilized for the prediction of bacteriophages (Zhou et al., 2011). Typing of plasmids was performed based on a previous description (Lam et al., 2023). The plasmid structure was visualized using DNAPlotter (https://www.sanger.ac.uk/tool/dnaplotter/) (Carver et al., 2009). Plasmid comparisons were conducted using the Circoletto tool (http://tools.bat.infspire.org/circoletto/) (Darzentas, 2010). Similar plasmids in *Acinetobacter* spp., *Providencia rettgeri*, and *Klebsiella pneumoniae* were tracked using the BacWGSTdb server (Marquez-Ortiz et al., 2017; Feng et al., 2021).

## Results

### Genome annotations and subsystem categories

**Figure 1:**
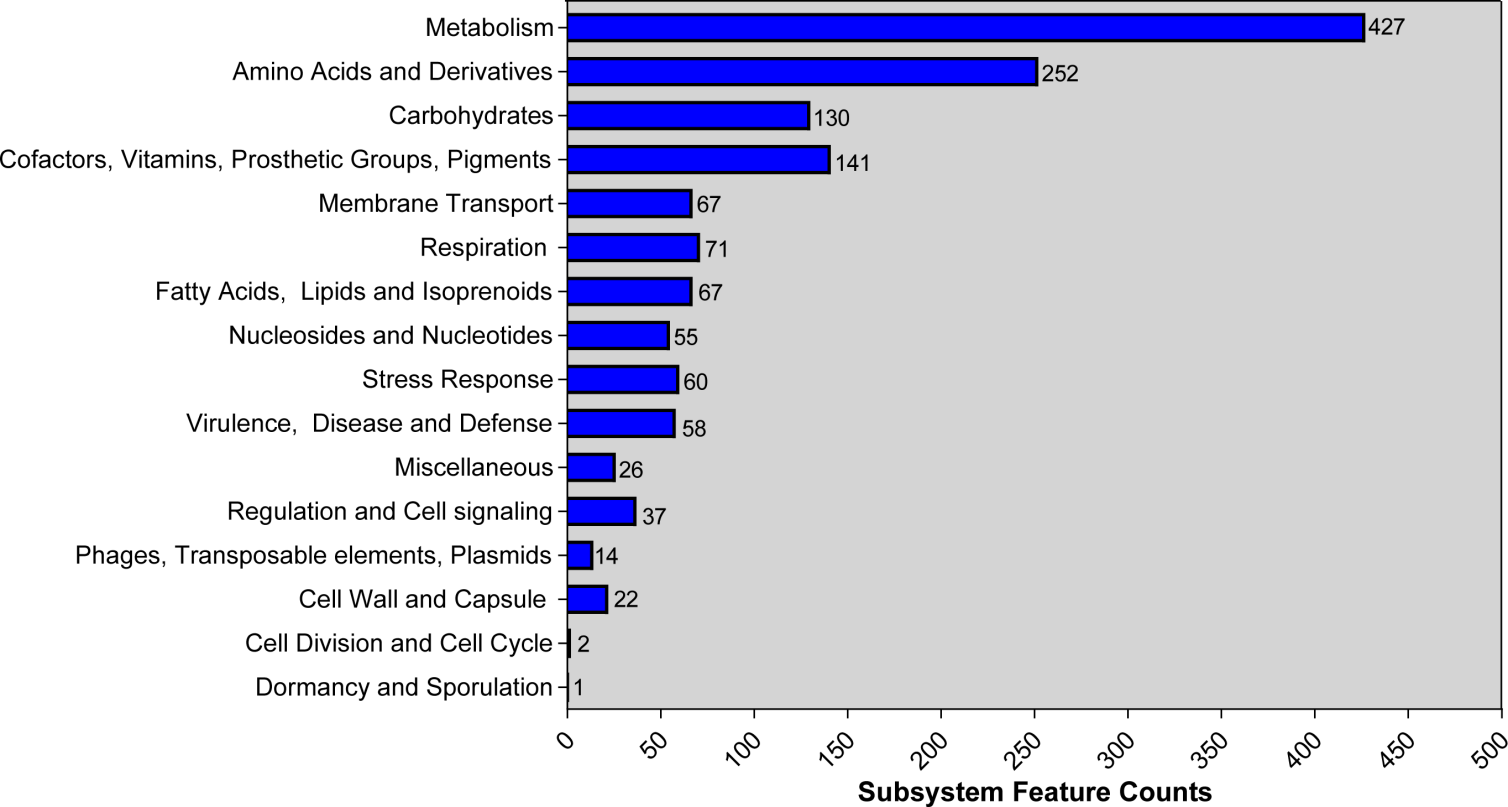
RAST annotation of *A. johnsonii* AYTCM strain. The number of each subsystem category is shown on the right of column.

Genome was annotated using PGAP and RAST. Based on PGAP annotation, there are 3,980 genes in total, of which 3,731 are protein-coding genes, 136 are pseudo genes, and the remaining 113 are predicted RNA-coding genes. Compared with the PGAP server, 4,182 genes, including 109 RNA-coding genes, belonged to 293 subsystems when annotated using RAST. The statistics of the subsystem is shown (**Figure 1**). Most of them belonged to metabolism (427), amino acids and derivatives (252), and carbohydrates (130). Additionally, 14 CDS were sorted into “Phages, transposable elements, plasmids” and only 2 and 1 CDS belonged to “cell division and cell cycle” and “dormancy and sporulation,” respectively. Functional comparison showed that most subsystems were metabolism among three *A. johnsonii* strains. However, a huge difference was found in “Phages, Prophages, Transposable elements, Plasmids”. There are two CDS that belonged to “Phages, Prophages, Transposable elements, Plasmids” in *A. johnsonii* C6 and MB44 strains. However, 14 subsystems of this function were identified in *A. johnsonii* AYTCM strain.

### MICs, antimicrobial resistance, and virulence profiles

Antimicrobial susceptibility testing revealed that *A. johnsonii* AYTCM strain possessed a multidrug-resistant (MDR) profile and the meropenem and imipenem MICs are all >128 mg/L. Furthermore, it exhibited resistance to ceftazidime (>128 mg/L), ciprofloxacin (>32 mg/L), and cefoperazone/sulbactam (128 mg/L) but still remained susceptible to tigecycline (1 mg/L) and cefiderocol (<0.03 mg/L). The MICs of colistin and amikacin are 2 mg/L and 32 mg/L, respectively, which were defined as intermediate.

Analysis of the genome of *A. johnsonii* AYTCM strain revealed that, in addition to co-harboring chromosomal *bla*_OXA-652_ and *aadA27*, a series of other antibiotic resistance genes were identified, including *bla*_OXA-58_, *bla*_NDM-1_ *, bla*_PER-1_, msr*(E)*, mph*(E)*, aac(3)-IId, aph(3′)-VIa, sul1, arr-3, qnrVC6*, ble-MBL*, *aph(3′)-VI, tet*(39), *sul2*, and *bla*_MCA_ (**Table 1**). However, only two virulence factors, two-component regulatory system *bfmRS* involved in Csu expression and *lpxC*-encoding lipopolysaccharide (LPS), were found in AYTCM strain.

**Table 1:** Molecular characterization of the genome of *A. johnsonii* AYTCM strain.

Genome	Replicon	Size (bp)	GC content	Resistance genes	Accession numbers
Chromosome	ND	3,567,832	41.60%	*bla*_OXA-652_, *aadA27*	CP121776
pAYTCM-1	ND	378,197	39.93%	*bla*_OXA-58_, msr*(E)*, mph*(E)*, aac(3)-IId, aph(3′)-VIa, sul1, arr-3, qnrVC6*, bla*_PER-1_	CP121777
pAYTCM-2	ND	44,599	36.89%	ND	CP121778
pAYTCM-3	ND	41,087	38.32%	*bla*_NDM-1_ *, ble-MBL*, *aph(3′)-VI*	CP121779
pAYTCM-4	ND	22,357	35.15%	msr*(E)*, mph*(E)*	CP121780
pAYTCM-5	ND	13,499	35.78%	ND	CP121781
pAYTCM-6	ND	8,636	35.47%	*tet*(39)	CP121782
pAYTCM-7	Aci1	7,579	38.88%	*sul2*	CP121783
pAYTCM-8	ND	6,289	34.38%	*bla*_MCA_	CP121784
pAYTCM-9	ND	6,147	37.17%	ND	CP121785
pAYTCM-10	ND	4,135	42.44%	ND	CP121786
pAYTCM-11	ND	2,356	36.54%	ND	CP121787

ND, not detected.

### ANI, core-genome phylogeny, lipooligosaccharide outer core, and capsular polysaccharide (KL)

According to the ANI analysis, the result showed that 95.82% two-way ANI between *A. johnsonii* AYTCM and *A. johnsonii* C6 and 95.86% ANI were found between *A. johnsonii* AYTCM and *A. johnsonii* MB44 and only 79.89% two-way ANI between *A. johnsonii* AYTCM and *A. baumannii* ATCC 17978. Core-genome phylogeny analysis showed a close genetic relationship among *A. johnsonii* AYTCM, C6, and MB44 strains. However, a huge diversity was observed among *A. baumannii*, *A. pittii*, and other *A. seifertii* strains based on the phylogenetic tree (**Figure S2A**). Similar results of SNP difference are shown in **Figure S2B**.

*Kaptive* revealed that AYTCM strain contains OC locus 1c (OCL-1c), matching the 92.01% nucleotide identity. The K locus in *A. johnsonii* AYTCM strain is KL19, to which it matches with an overall nucleotide identity of 72.75%.

### Transfer ability and stability of plasmids in the *A. johnsonii* AYTCM strain

Mating assays were performed to explore the transfer ability of *bla*_NDM-1_, *bla*_OXA-58_, and *bla*_PER-1_ genes; results showed that only *bla*_NDM-1_ could transfer to the recipient strain. The stability assays revealed that all three resistance genes were quite stable even after 70 passages under antibiotics-free conditions.

### Genome characterization of the chromosome and 11 plasmids

Hybrid assembly of the short and long reads generated a 3,567,832-bp size circular chromosome with a GC content of 41.60% (**Table 1**). One intrinsic resistance gene, *bla*_OXA-652_, was identified in the chromosome. Of note, *A. johnsonii* AYTCM strain carries 11 plasmids, namely, pAYTCM-1 to pAYTCM-11, with sizes between 2,356 bp and 378,197 bp and GC contents ranging from 34.38% to 42.44% (**Table 1**). Apart from pAYTCM-2, pAYTCM-5, pAYTCM-9, pAYTCM-10, and pAYTCM-11, various kinds of resistance genes were found in other plasmids. Analysis of *rep* genes showed that only pAYTCM-7 possessed one identified name with Aci1.

### Genetic context characterization of pAYTCM-1 multidrug-resistant plasmid

**Figure 2:**
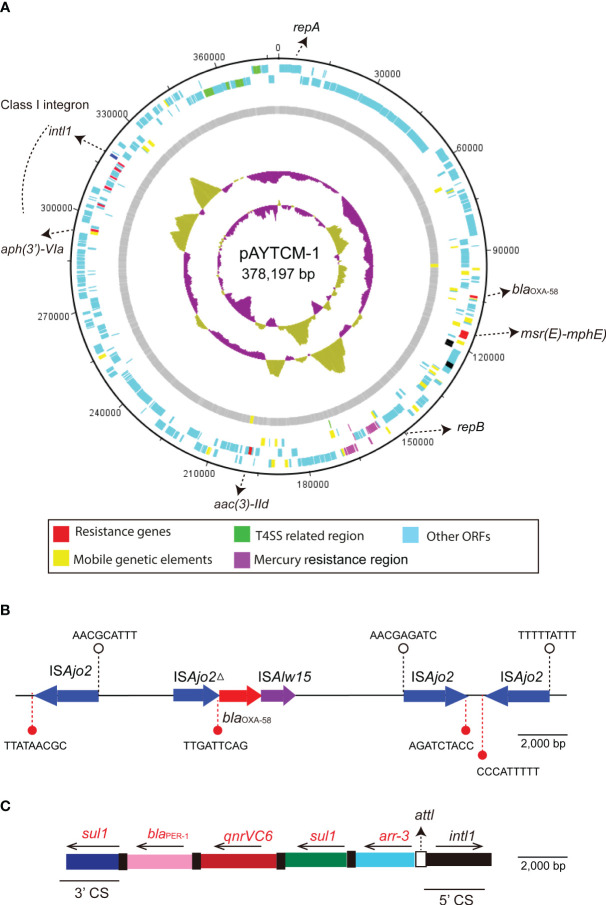
Circular map and genetic environment of the pAYTCM-1 plasmid. **(A)** Circular map of the pAYTCM-1 plasmid. Different filled boxes indicate various open reading frames (ORFs). The GC content and GC skew are shown in the inner rings. Resistance genes (red filled boxes), mobile genetic elements (yellow filled boxes), T4SS region (green filled boxes), and mercury resistance region (purple filled boxes). Light blue represents other ORFs. **(B)** Genetic environment of the *bla*_OXA-58_ gene. The red filled arrow indicates the position of the *bla*_OXA-58_ gene. Blue filled arrows indicate IS*Ajo2* and IS*Ajo2Δ*. Purple filled arrow indicates IS*Alw15*. Arrows’ directions indicate the ORF directions. 9-bp target site duplications (TSD) are shown upstream and downstream of IS*Ajo2* and IS*Ajo2Δ* using white or red filled circles, respectively. **(C)** Structure of the class 1 integron containing *bla*_PER-1_. *intl1* is shown as a black filled box. attl is shown as a white filled box. The 5′ conserved segment (5′ CS) and 3′ CS of class 1 integron are labeled. The various kinds of resistance genes were shown as different colors with the names labeled above with the orientation indicated by thin black arrows.

**Figure 3:**
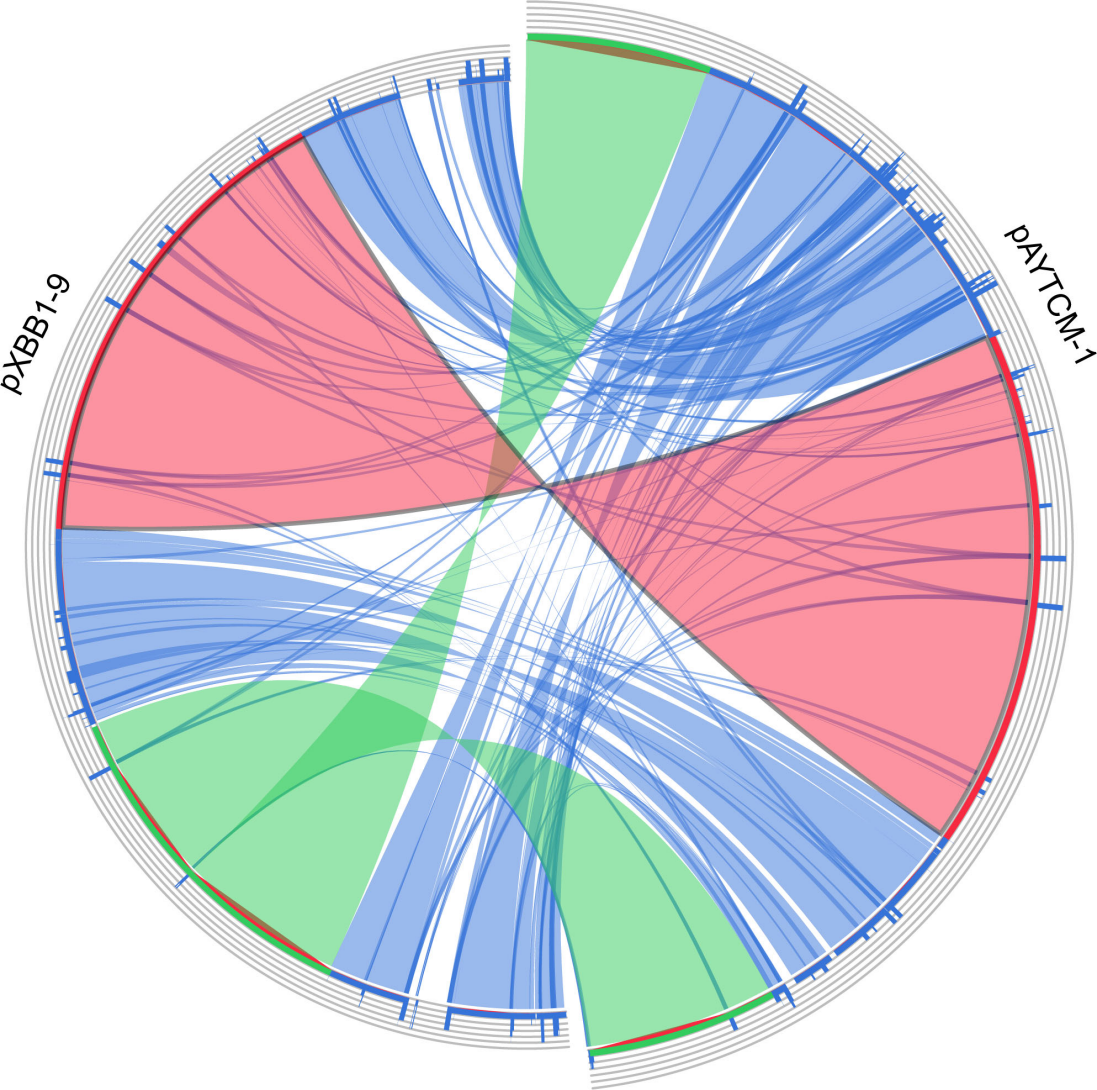
Plasmid comparison with pXBB1-9 using Circoletto. Ribbons represent the alignments produced by BLAST, their width the alignment length, and the colors the alignment bitscore in four quartiles: blue for the first 25% of the maximum bitscore, green for the next 25%, orange for the third, and finally red for the top bitscores of between 75% and 100%.

pAYTCM-1 is a huge 378,197-bp multidrug-resistant plasmid with an average GC content of 39.93%. It comprises different regions, including type IV secretion system (T4SS) region, class 1 integron region, and mercury resistance region (**Figure 2A**). *bla*_OXA-58_ and *bla*_PER-1_ genes were located in the pAYTCM-1 plasmid. Concerning the genetic context of *bla*_OXA-58_, three intact and one truncated IS*Ajo2* were located upstream or downstream. 9-bp TSD sequences were observed in the upstream and downstream of IS*Ajo2* genetic elements. Nevertheless, the TSD sequences were all different (**Figure 2B**). Importantly, a complex class 1 integron complex consisted of a 5′ conserved segment (5′ CS) and 3′ CS, which was found to carry sul1, arr-3, qnrVC6*, and bla*_PER-1_ cassettes (**Figure 2C**). Of note, 10 XerC and XerD-like binding sites (p*dif* sites) were found in the pAYTCM-1 plasmid (**Table 2**). In addition, no *oriT* was identified in the pAYTCM-1 plasmid and no transconjugants were obtained via conjugation. Moreover, results of the Circoletto tool showed that there were many similar segments between pAYTCM-1 and pXBB1-9 (GenBank accession number: CP010351) plasmids (**Figure 3**). Genetic structure comparison revealed that 98% coverage and 99.91% identity were identified between pAYTCM-1 and pXBB1-9 plasmids, which was found in the *A. johnsonii* XBB1 isolate from a hospital sewage in 2010 in Chengdu, western China.

**Table 2:** p*dif* sites of the pAYTCM-1 plasmid.

Name	Start	End	Left arm	Center	Right arm	Site
pdif1	97,630	97,657	ATTTCGTATAA	GGTGTA	TTATGTTAATT	C|D
pdif2	99,839	99,866	GATTCGTATAA	GGTGTA	TTATGTTAATT	D|C
pdif3	101,969	101,996	ATTTAACATAA	TGGCTG	TTATACGAAAC	C|D
pdif4	106,222	106,249	ATTTTGTATAA	GGTGTA	TTATGTTAATT	D|C
pdif5	107,789	107,816	ATTTAACATAA	TGGGCG	TTATACGAAAA	C|D
pdif6	108,640	108,667	ACTTCGCATAA	CGCCCA	TTATGTTAATT	D|C
pdif7	109,282	109,309	ACTTAACATAA	TGGCGG	TTATACGAAAT	C|D
pdif8	110,501	110,528	ATTTAACATAA	TGGCTG	TTATGCGAACG	D|C
pdif9	117,181	117,208	ATTTAACATAA	AATTTC	TTATGTGAAGT	C|D
pdif10	260,147	260,174	AATCTAGATAA	TTAGCA	ATATACGATAT	D|C

### Genetic features of *bla*_NDM-1_-carrying plasmid pAYTCM-3

**Figure 4:**
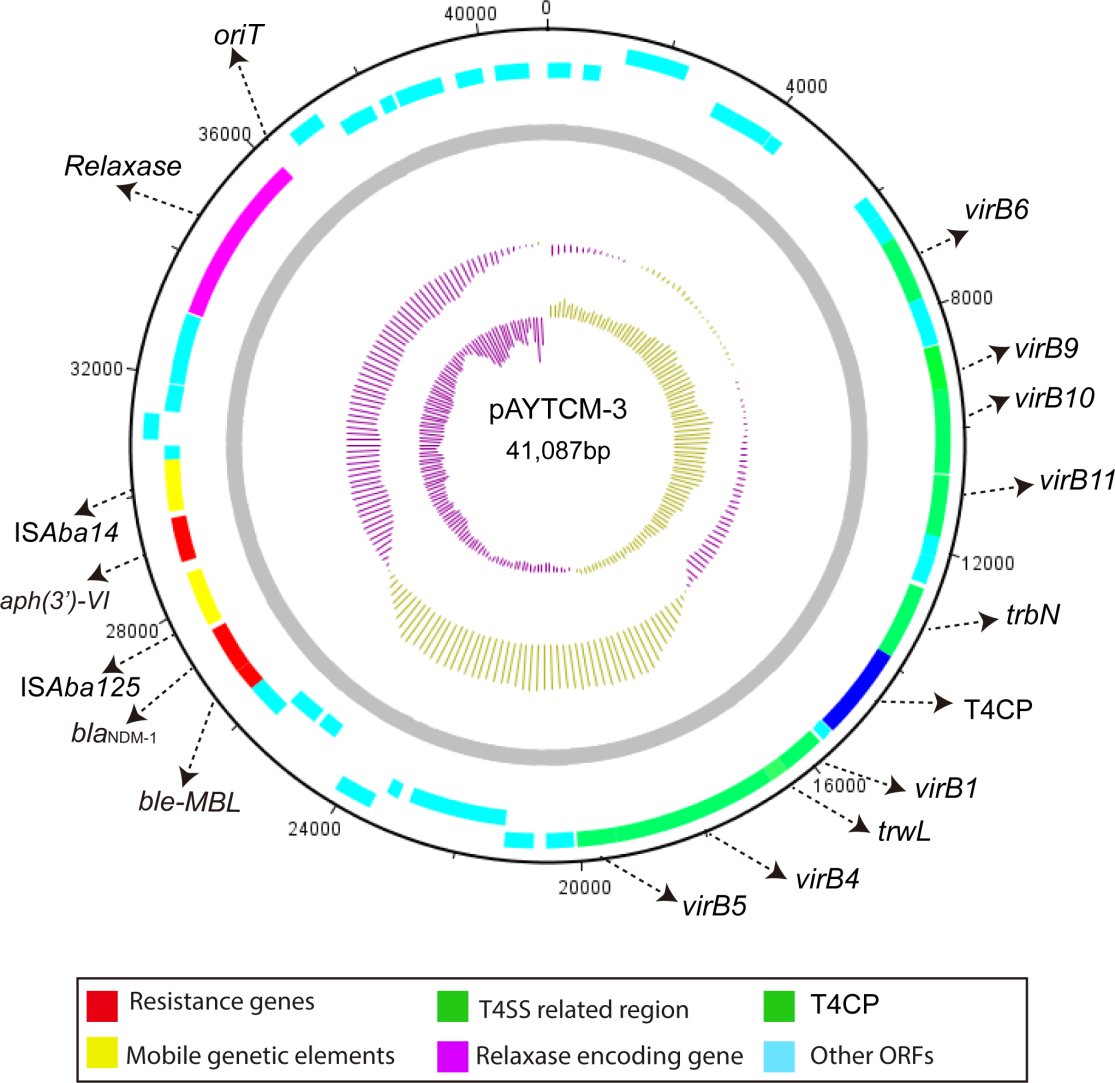
Circular map of pAYTCM-3. Different filled boxes indicate various open reading frames (ORFs). The GC content and GC skew are shown in the inner rings. Resistance genes are shown as a red filled box. Mobile genetic elements are shown as a yellow filled box. The T4SS region and T4CP are indicated as a green filled box. The relaxase-encoding gene is shown in the purple filled box. Light blue represents other ORFs. Moreover, the position of *oriT* was also labeled.

The *bla*_NDM-1_ carbapenem gene was located in 41,087 plasmids with the GC content of 38.32%. Genetic context analysis revealed that *bla*_NDM-1_ was IS*Aba14*-*aph(3′)-VI*-IS*Aba125*-*bla*_NDM-1_-*ble-MBL* (**Figure 4**). Moreover, a T4SS region, T4CP, a gene encoding relaxase, and a 38-bp *oriT* region (AGGGATTCATAAGGGAATTATTCCCTTATGTGGGGCTT) were identified. pAYTCM-3 could transfer to *E. coli* J53 via conjugation.

### Prophage regions in the chromosome

**Figure 5:**
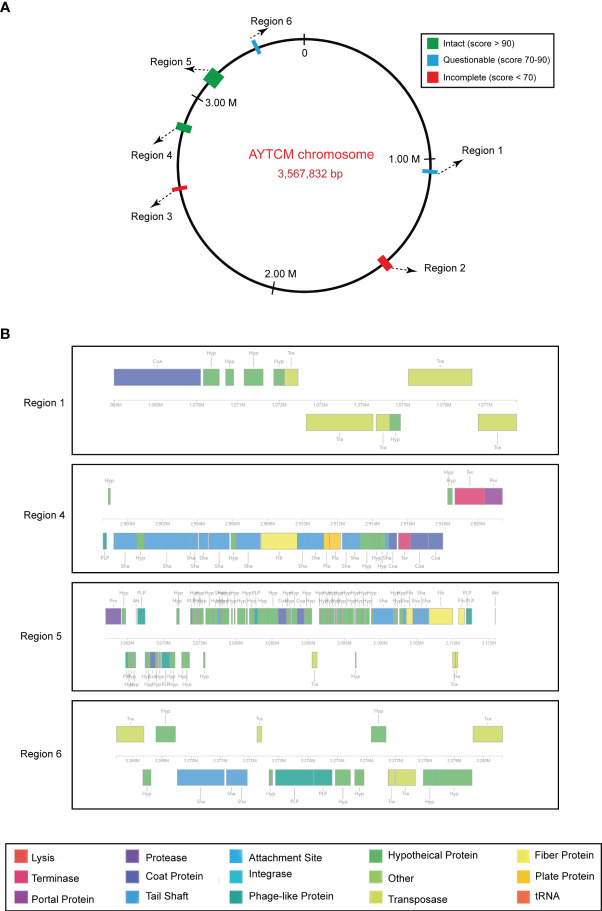
Predicted prophage regions within the *A johnsonii* AYTCM chromosome. **(A)** Six prophage regions positions in the chromosome. Green filled boxes mean the intact prophage regions (score > 90), filled boxes mean the questionable prophage regions (score 70–90), filled boxes mean the incomplete prophage regions (score < 70). **(B)** Structure of two intact and two questionable prophage regions. Genes are colored based on predicted functions.

Prophage regions were predicted by the PHASTER tool; results showed two intact, two questionable, and two incomplete regions in the chromosome (**Figure 5A**). Based on the PHASTER tool, regions 4 and 5 were predicted to be intact due to the score of >90. In addition, regions 1 and 6 were classified as questionable due to the scores of 70–90. However, regions 2 and 3 were shown as incomplete due to the low scores. Gene functions of the two intact and two questionable prophage regions are shown, including attachment, phage integration, and cell lysis (**Figure 5B**).

### Track and characteristics of closely related plasmids in the public database

To track the closely related plasmids from different countries, a wide search was performed via the BacWGSTdb server. Data showed that pAYTCM-1 was similar to pXBB1-9, pOXA23_010062, pOXA58_010030, and pAcsw19-2 plasmids (**Table 3**). Their sizes are all >300 kb, and they were collected from the strains of sewage in China. However, the species were various, including *A. johnsonii*, *A. wuhouensis*, and *A. defluvii*.

**Table 3:** Track of similar plasmids using the BacWGSTdb database.

Plasmid name	Accession number	Size (bp)	Species	Host	Source	Country	Year	Antimicrobial resistance genes
pXBB1-9	NZ_CP010351.1	398,857	*A. johnsonii*	–	Sewage	China	2010	*arr-3*, *aac(3)-IId*, *aac(6′)-Ib*, *aph(3″)-Ib*, *aph(3′)-*VIa, *aph(6)-Id*, *bla*_OXA-58_, *bla*_PER-1_, *mph*(E), *msr*(E), *sul1*, *tet*(Y)
pOXA23_010062	NZ_CP033130.1	311,749	*A. wuhouensis*	–	Sewage	China	2015	*aac(3)-IId*, *aph(3′)-*VIa, *bla*_OXA-23_, *bla*_OXA-58_, *mph*(E), *msr*(E)
pOXA58_010030	NZ_CP029396.2	355,358	*A. defluvii*	–	sewage	China	2015	*arr-3*, *aac(6′)-Ib3*, *aph(3′)-*VIa, *bla*_OXA-58_, *mph*(E), *msr*(E), *sul1*, *sul2*
pAcsw19-2	NZ_CP043309.1	351,885	*A. johnsonii*	–	sewage	China	2019	*aph(3′)-VIa*, *bla*_NDM-1_, *bla*_OXA-58_, *mph*(E), *msr*(E)
pNDM-JN01	KM210086.1	41,084	*A. lwoffii*	Homo sapiens	Feces	China	2023	*aph(3′)-VI*, *bla*_NDM-14_
pAB17	MT002974.1	41,087	*A. baumannii*	Homo sapiens	–	Brazil	2023	*aph(3′)-VI*, *bla*_NDM-1_
unnamed2	NZ_CP027532.1	41,087	*A. baumannii*	–	–	USA	2018	*aph(3′)-VI*, *bla*_NDM-1_
p4TQ-NDM	NZ_CP045130.1	41,086	*A. indicus*	Cow	Feces	China	2017	*aph(3′)-VI*, *bla*_NDM-1_
pNDM-AP	KJ003839.1	39,364	*A. pittii*	Homo sapiens	Blood	China	–	*aph(3′)-VI*, *bla*_NDM-1_
pSU1805NDM	LC483156.1	41,022	*A. pittii*	–	Hospital environment	Japan	–	*aph(3′)-VI*, *bla*_NDM-1_
pIEC38057	MK053934.1	41,085	*A. nosocomialis*	–	Blood	Brazil	2016	*aph(3′)-VI*, *bla*_NDM-1_
pNDM-0285	NZ_CP026127.1	39,359	*A. baumannii*	–	–	USA	2016	*aph(3′)-VI*, *bla*_NDM-1_
p18TQ-NDM	NZ_CP045133.1	40,439	*A. indicus*	Cow	Feces	China	2017	*aph(3′)-VI*, *bla*_NDM-1_
p23TQ-NDM	NZ_CP045197.1	41,393	*A. indicus*	Cows	Feces	China	2017	*aph(3′)-VI*, *bla*_NDM-1_
pNDM-AB	NC_020818.1	47,098	*A. baumannii*	Pig	Lung	China	–	*aph(3′)-VI*, *bla*_NDM-1_, *mph*(E), *msr*(E)
pXM1	AMXH01000087.1	47,274	*A. pittii*	Homo sapiens	Sputum	China	2010	*aph(3′)-VI*, *bla*_NDM-1_
pNDM-BJ01	NC_019268.1	47,274	*A. lwoffii*	–	–	China	2011	*aph(3′)-VI*, *bla*_NDM-1_
pNDM-BJ02	NC_019281.1	46,165	*A. lwoffii*	–	–	China	2011	*aph(3′)-VI*, *bla*_NDM-1_
p6200-47.274kb	NZ_CP010399.1	47,274	*A. baumannii*	Homo sapiens	Bodily fluid	Colombia	2012	*aph(3′)-VI*, *bla*_NDM-1_
pAbNDM-1	NC_019985.2	48,368	*A. baumannii*	–	–	China	–	*aph(3′)-VI*, *bla*_NDM-1_
p6411-9.012kb	NZ_CP010370.2	47,274	*A. nosocomialis*	Homo sapiens	Excreted bodily substance	Colombia	2012	*aph(3′)-VI*, *bla*_NDM-1_
pAhaeAN54e	NZ_CP041229.1	45,460	*A. haemolyticus*	Homo sapiens	Peritoneal dialysis fluid	Mexico	2016	*aph(3′)-VI*, *bla*_NDM-1_
pNDM-Iz4b	NC_025000.1	46,570	*A. lwoffii*	Homo sapiens	–	China	–	*aph(3′)-VI*, *bla*_NDM-1_
pNDM1_060092	NZ_CP035935.1	48,560	*A. cumulans*	–	Sewage	China	2018	*aph(3′)-VI*, *bla*_NDM-1_
pNDM-40-1	NC_023322.1	45,826	*A. bereziniae*	Homo sapiens	Pus	India	2005	*aph(3′)-VI*, *bla*_NDM-1_
pNDM1_010005	NZ_CP032132.1	39,357	*A. chinensis*	–	Sewage	China	2015	*aph(3′)-VI*, *bla*_NDM-1_

"-" means unknown.

Concerning the closely related plasmids of pAYTCM-3, results showed that hosts, also carrying the *bla*_NDM-1_-related plasmid, were collected from several different sources, including feces, blood, sputum, pus, sewage, and hospital environment, from 2005 to 2023. These *bla*_NDM-1_-harboring plasmids were all collected in *Acinetobacter* spp. and not in *P. rettgeri* and *K. pneumoniae*. Their hosts were isolated from various countries, such as China, USA, Japan, Brazil, and Mexico.

## Discussion

Emergence of carbapenemase-producing *Acinetobacter* spp. has become dominant in several countries, and it is being increasingly considered a quite important nosocomial pathogen and poses a huge challenge to the healthcare setting (Mohd Rani et al., 2017). Class D β-lactamases (mainly OXA-23), commonly named as OXA, are responsible for carbapenem resistance in *Acinetobacter* spp. species (Zong et al., 2020). However, the reports of other β-lactamases (e.g., NDM-1) are relatively rare, especially for those with a high resistance level. In this work, NDM-1 and OXA-58 were found in our strain, which leads to a high-level carbapenem resistance. To promote better understanding regarding the genomic features of our *A. johnsonii* strain, whole-genome sequencing and further RAST software were used to classify the different CDS into subsystems based on their function. Consistent with other *A. pittii* strains, the majority of the CDS belong to the function of “Metabolism” (Chapartegui-Gonzalez et al., 2022).

Mobile genetic elements (MGEs), including ISs, integrons, and transposons, play a particularly important role in the movement and dissemination of resistance genes (Gorbunova et al., 2021). Concerning the acquisition of the *bla*_OXA-58_ gene, many copies of IS*Ajo2* were identified in the pAYTCM-1 plasmid and located upstream and downstream of *bla*_OXA-58_. Nevertheless, considering the various 9-bp TSD sequences of IS*Ajo2*, we failed to find direct evidence to conclude that *bla*_OXA-58_ was embedded into the plasmid via different IS*Ajo2.* Interestingly, XerC/XerD-like recombinase sites (p*dif* sites) were considered as a new approach for the transfer of carbapenem resistance genes, such as *bla*_OXA-24_, *bla*_OXA-72_, and *bla*_OXA-58_ (Merino et al., 2010; Kuo et al., 2016; Liu et al., 2021). Here, 10 p*dif* sites were identified in the pAYTCM-1 plasmid. Furthermore, we observed that the *bla*_OXA-58_ gene was flanked by two p*dif* sites. Consequently, the *bla*_OXA-58_ gene might have been introduced by p*dif* site-mediated specific recombination. This is consistent with previous research (Feng et al., 2016). Moreover, considering the high coverage and identity with pXBB1-9 (Feng et al., 2016), we deduced that the pAYTCM-1 plasmid may come from a hospital environment-related *A. johnsonii* isolate XBB1 strain and underwent slight evolution. Another finding in this study is that *bla*_PER-1_ was also located in the pAYTCM-1 plasmid. Liu et al. reported that the production of PER-1 in *A. baumannii* is the key mechanism of cefiderocol resistance (Liu et al., 2022b). However, the MIC of cefiderocol was low and considered as susceptible in our *A. johnsonii* AYTCM strain. We inferred that the resistance in *A. baumannii* was caused by species specificity.

In our previous study, we reported that *bla*_NDM-1_ was located in the chromosome, which was mediated by two IS*Aba125*-based Tn*125* composite transposons, highlighting the importance of Tn*125*-mediated transfer of *bla*_NDM-1_ resistance determinants (Tian et al., 2022). However, we could not find the composite Tn*125* transposon in the *A. johnsonii* AYTCM strain due to that only one copy of IS*Aba125* was identified. In addition, two studies from Krahn et al. and Abouelfetouh et al. showed that prophages may play a key role in the carbapenem resistance genes, such as *bla*_NDM-1_ and *bla*_OXA-23_ (Krahn et al., 2016; Abouelfetouh et al., 2022). In addition, a study demonstrated the presence of resistance genes (including *mcr-1* and *vanA*) in the phage fraction and its role on the acquisition and transfer of these resistance genes (Pires et al., 2023). However, the *bla*_NDM-1_ gene is not part of any of the prophages. Hence, the relationship of these prophages and the *bla*_NDM-1_ gene should be further confirmed through induced experiments. Concerning the *bla*_NDM-1_-harboring plasmids, we discovered that they were located in diverse sources and hosts and in various countries. These data indicated that a wide spread of *bla*_NDM-1_-bearing plasmids has occurred all over the world. However, these plasmids usually transferred among different *Acinetobacter* species. Concerning the various resistance plasmids in *A. johnsonii* AYTCM strain, it is revealed that our strain has great potential to capture plasmids that contribute to its resistance. Since our strain is of patient origin, there may be a great possibility that this strain will emerge and further spread between patients and the environment in the hospital. More importantly, Lam et al. reported that the Aci1 plasmid usually was found in extensively and pan-resistant *A. baumannii* isolates which belong to global clones GC1 and GC2 (Lam et al., 2023). Here, the Aci1 plasmid has been identified in *A. johnsonii* strain, further suggesting that the Aci1 plasmid has transferred among various *Acinetobacter* species.

Apart from resistance determinants, virulence factors should also be paid attention in bacteria. However, the low content of virulence factors in *A. johnsonii* AYTCM strain is in clear contrast to the high number of resistance genes. Thus, in the surveillance of *A. johnsonii*, researchers should probably pay more attention to the antimicrobial resistance when compared with virulence. This is a different aspect from the hypervirulent carbapenem-resistant *K. pneumoniae* (Pu et al., 2023).

## Conclusion

This study is the first comprehensive description for the complete genome characteristics of a carbapenem-resistant *A. johnsonii*, co-producing NDM-1, OXA-58, and PER-1 from a patient source. The *A. johnsonii* isolate AYTCM carried 11 plasmids, which revealed great genome plasticity for this species, which possesses huge potential to capture resistance plasmids. Moreover, the Aci1 plasmid has been identified in *A. johnsonii* strain using the current plasmid typing system. However, other eight plasmids failed to type. Therefore, the *rep* genes for the plasmid typing system need to be further explored. Early surveillance of this kind of carbapenem-resistant isolate is warranted to avoid the extensive spread of this high-risk clone in the healthcare setting.

## Data availability statement

The original contributions presented in the study are publicly available. This data can be found here: https://www.ncbi.nlm.nih.gov/; PRJNA953498.

## Ethics statement

This study was approved by the local Ethics Committees of the Hospital with a waiver of informed consent since this study mainly focused on bacterial genome and the retrospective nature of the study.

## Author contributions

CT and JS designed the experiments, analyzed the data, and wrote the initial manuscript. CT, LR, DH, SW, LF, YZ, and YB performed the majority of the experiments. JS collected the bacteria. XF, TM, and JY supervised this study and reviewed and edited the paper. All authors read and approved the final version of the manuscript.
