# Supplementary material for: Complete genetic characterization of carbapenem-resistant Acinetobacter johnsonii, co-producing NDM-1, OXA-58, and PER-1 in a patient source
Source: Front Cell Infect Microbiol. 2023 Aug 25;13:1227063. doi: 10.3389/fcimb.2023.1227063 (PMC10486904; doi:10.3389/fcimb.2023.1227063)
Supplement: Supplementary file 1 [file DataSheet_1.docx]

**
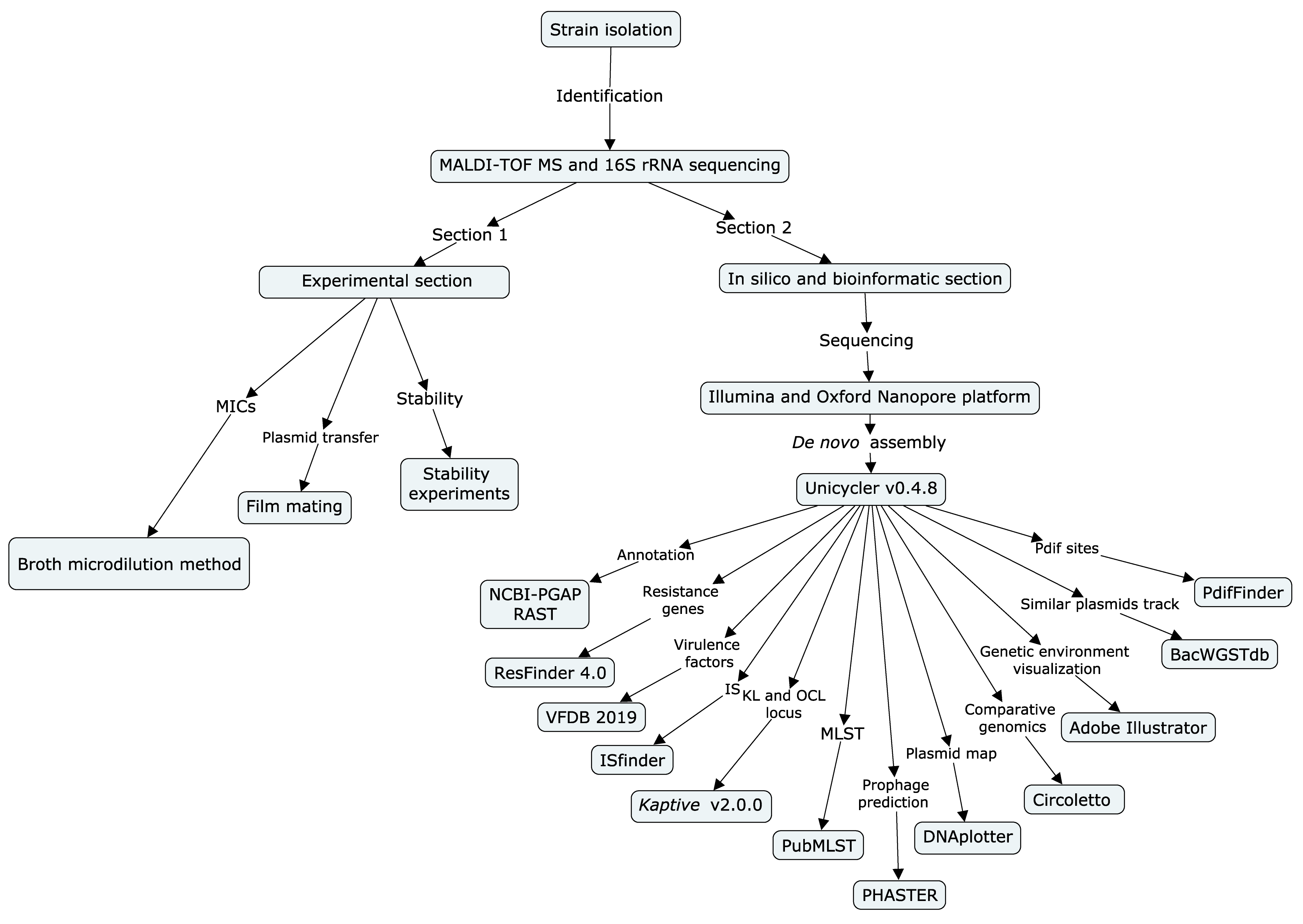
**

**Figure S1.** Flow chart of this study. A flow chart was built to show experiments and bioinformatic procedures of this study using CmapTools v6.04 (https://cmap.ihmc.us). The left panel was the experimental section, including MICs measurement, plasmids transfer assays and plasmids stability experiments. The right panel was the bioinformatic analysis section, including sequence annotation, resistance and virulence genes analysis, MLST, IS, KL, OCL and related plasmids structure and comparative study.


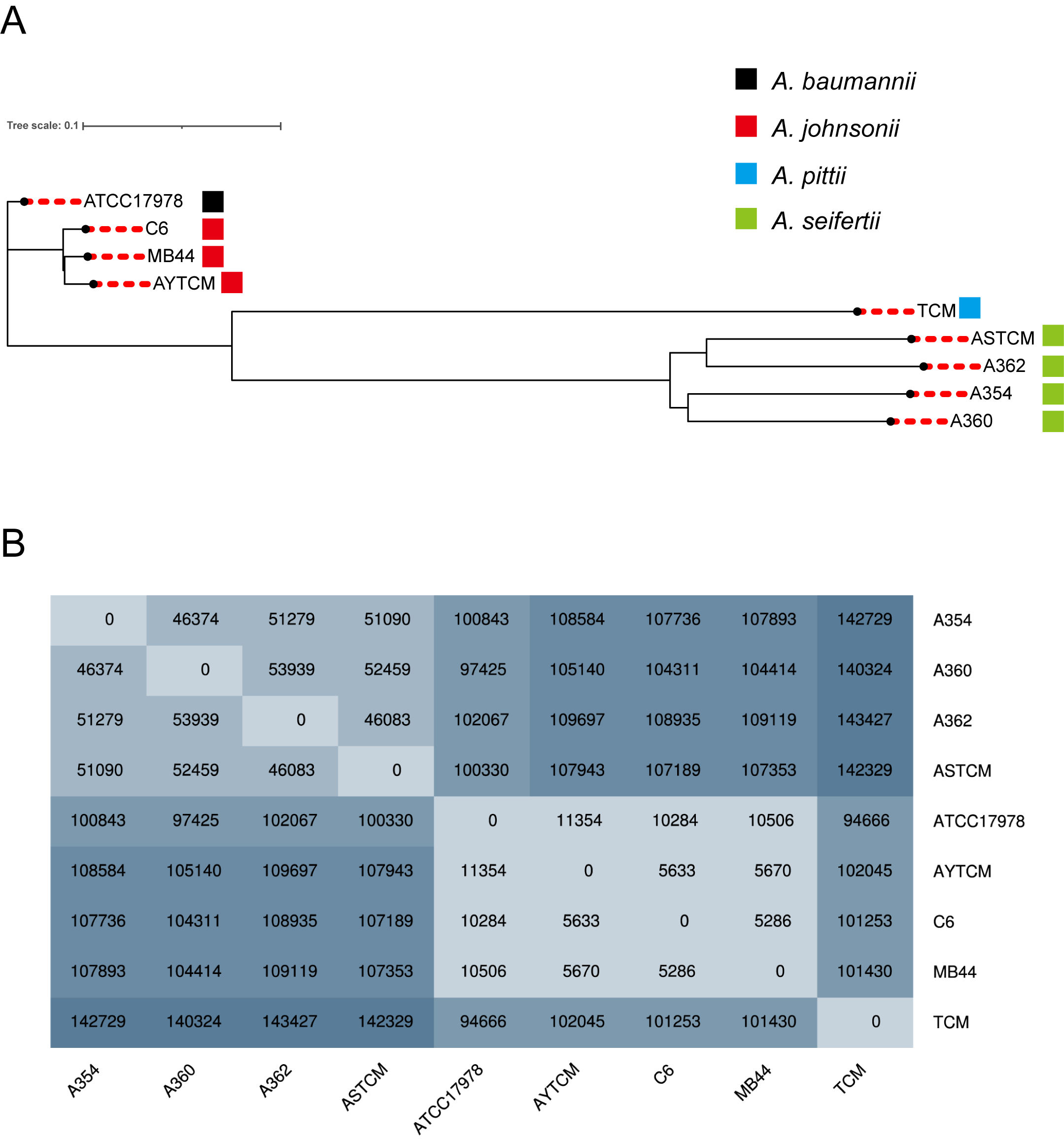


**Figure S2.** Phylogenetic analysis and heatmap of the SNP count matrix. (A) A core-genome phylogeny was built with *A. johnsonii*, *A. baumannii*, *A. pittii* and *A. seifertii* with BacWGSTdb tool (http://bacdb.cn/BacWGSTdb/Tools.php). Then GenBank accession no. are as follow: *A. johnsonii* AYTCM (this study); *A. johnsonii* C6 (accession no. FUUY00000000); *A. johnsonii* MB44 (accession no. accession no. LBMO00000000); *A. baumannii* ATCC17978 (accession no. NZ_CACVBA010000001-NZ_CACVBA010000004); *A. pittii* TCM (accession no. CP095407-CP095411); *A. seifertii* ASTCM (accession no. JARLUF000000000); *A. seifertii* A354 (accession no. LFZQ01); *A. seifertii* A360 (accession no. LFZR01); *A. seifertii* A362 (accession no. LFZS01). (B) SNP difference was shown in the boxes. Data was visualized using BacWGSTdb server.
